# The utility of MRI histogram and texture analysis for the prediction of histological diagnosis in head and neck malignancies

**DOI:** 10.1186/s40644-019-0193-9

**Published:** 2019-02-04

**Authors:** Noriyuki Fujima, Akihiro Homma, Taisuke Harada, Yukie Shimizu, Khin Khin Tha, Satoshi Kano, Takatsugu Mizumachi, Ruijiang Li, Kohsuke Kudo, Hiroki Shirato

**Affiliations:** 1 Department of Diagnostic and Interventional Radiology, Hokkaido University Hospital, N14 W5, Kita-Ku, Sapporo, 0608638 Japan; 2 Department of Otolaryngology-Head and Neck Surgery, Hokkaido University Graduate School of Medicine, N15 W7, Kita-Ku, Sapporo, 0608638 Japan; 3 Department of Radiation Medicine, Hokkaido University Graduate School of Medicine, N15 W7, Kita-Ku, Sapporo, 0608638 Japan; 4 The Global Station for Quantum Medical Science and Engineering, Global Institution for collaborative research and education, N15 W8, Kita-Ku, Sapporo, 0608638 Japan; 5 Department of Radiation Oncology, Stanford University, 875 Blake Wilbur Drive, Stanford, 94305-5847 CA USA

**Keywords:** Histogram analysis, Texture analysis, Head and neck squamous cell carcinoma, Histological grade, Malignant lymphoma, Differentiation

## Abstract

**Background:**

To assess the utility of histogram and texture analysis of magnetic resonance (MR) fat-suppressed T2-weighted imaging (Fs-T2WI) for the prediction of histological diagnosis of head and neck squamous cell carcinoma (SCC) and malignant lymphoma (ML).

**Methods:**

The cases of 57 patients with SCC (45 well/moderately and 12 poorly differentiated SCC) and 10 patients with ML were retrospectively analyzed. Quantitative parameters with histogram features (relative mean signal, coefficient of variation, kurtosis and skewness) and gray-level co-occurrence matrix (GLCM) features (contrast, correlation, energy and homogeneity) were calculated using Fs-T2WI data with a manual tumor region of interest (ROI).

**Results:**

The following significantly different values were obtained for the total SCC versus ML groups: relative mean signal (3.65 ± 0.86 vs. 2.61 ± 0.49), contrast (72.9 ± 16.2 vs. 49.3 ± 8.7) and homogeneity (2.22 ± 0.25 × 10^− 1^ vs. 2.53 ± 0.12 × 10^− 1^). In the comparison of the SCC histological grades, the relative mean signal and contrast were significantly lower in the poorly differentiated SCC (2.89 ± 0.63, 56.2 ± 12.9) compared to the well/moderately SCC (3.85 ± 0.81, 77.5 ± 13.9). The homogeneity in poorly differentiated SCC (2.56 ± 0.15 × 10^− 1^) was higher than that of the well/moderately SCC (2.1 ± 0.18 × 10^− 1^).

**Conclusions:**

Parameters obtained by histogram and texture analysis of Fs-T2WI may be useful for noninvasive prediction of histological type and grade in head and neck malignancy.

## Background

In head and neck malignancies, histopathological information is important for the determination of the exact diagnosis and for predicting the prognosis. Two types of diagnosis are frequent in head and neck malignancies: squamous cell carcinoma (SCC) and malignant lymphoma (ML) [1, 2].

The histopathological findings are the gold standard for the diagnoses and differentiation of SCC and ML. Additionally, in the pretreatment evaluation of SCC, the histological grade as well as the TNM stage classification has been described as an important prognostic factor related to the local control and the prediction of distant metastasis [3, 4]. However, in head and neck lesions, a surgical biopsy sometimes may miss the histological diagnosis, because tissue containing the tumor cells is not always obtained; peripheral inflammatory tissue is sometimes observed in the biopsy tissue [5]. In addition, a small tissue fraction by biopsy is not necessarily sufficient for the evaluation of the entire tumor’s characteristics in the diagnosis of SCC due to the issue of intra-tumor heterogeneity [6, 7]. Other supporting tools are thus needed for the precise histological diagnosis of head and neck malignancy.

Quantitative imaging methods such as histogram and texture analysis are now being investigated for the diagnosis, the prediction of treatment outcome, and the association with tumor genomic information in head and neck malignancies [8–11]. Such approach may be a noninvasive supporting tool for the histological diagnosis of head and neck malignancies.

We conducted the present study to assess the utility of histogram and texture analysis for the detailed histological diagnosis of head and neck malignancies. In this approach, we used the imaging data of magnetic resonance (MR) fat-suppressed T2 weighted images (Fs-T2WI), which is frequently used for noninvasive head and neck tumor imaging.

## Methods

### Patients

The protocol of this retrospective study was approved by our institutional review board (ID; 018–0038), and written informed consent was waived. We evaluated the cases of 67 patients with head and neck malignancy who were referred to our hospital and underwent MR scanning during the period from June 2009 to March 2018. All patients fulfilled the following inclusion criteria: (1) the patient was first diagnosed (not a recurrent case) histopathologically as having head and neck SCC or ML; (2) MR imaging (MRI) including axial Fs-T2WI was performed before any treatment, in accord with the specific MR equipment and parameters described below; and (3) the SCC patients’ histological grade (well, moderately or poorly differentiated SCC) was pathologically diagnosed. Patients with a primary site in the oropharynx, hypopharynx or oral cavity were chosen. Patients with a severe metal or motion artifact that seriously affected the image quality of the primary lesion were excluded. A primary lesion site in the larynx generally suffered from respiratory motion, and thus patients with this primary site were excluded. Nasal and paranasal sinus SCC patients were also excluded because the biological characteristics of these lesions are somewhat different from SCCs of the abovementioned primary sites [12]. In addition, some of the oropharyngeal SCC patients (*n* = 21) in the total study population received a diagnosis of human papillomavirus status from their histological samples by biopsy with the use of immunohistochemistry for p16.

### MR imaging protocol

All scanning was performed using a 3.0 Tesla MR-unit (Achieva TX; Philips Healthcare, Best, Netherlands) with a 16-channel neurovascular coil. MRI including the axial Fs-T2WI was performed to evaluate the primary tumor lesions. The sequence design and imaging parameters of the axial Fs-T2WI were as follows: a turbo spin-echo (TSE) sequence with fat suppression by spectral adiabatic inversion recovery (SPAIR) pulse, TR 4500 msec, TE 70 msec, TSE factor 9, FOV 240 × 240 mm, 400 × 512 acquisition matrix with 512 × 512 reconstruction by the zero-filling technique, slice thickness, 5 mm; inter-slice gap, 30%, total number of slices, 19. A part of the study population (nine well differentiated SCC, seven moderately differentiated SCC, five poorly differentiated SCC and 5 ML patients: total 26 patients) were also subjected to diffusion weighted imaging (DWI). The DWI acquisition used single-shot spin-echo echo-planar imaging with two b-values (0 and 1000 s/mm^2^) and the following parameters: TR, 4500 msec; TE, 64 msec; field of view (FOV), 230 × 230 mm; 64 × 64 matrix; slice thickness, 5 mm × 20 slices; voxel size 3.59 × 3.59 × 5.00 mm.

### Data analysis

#### Tumor ROI delineation

**Fig. 1:**
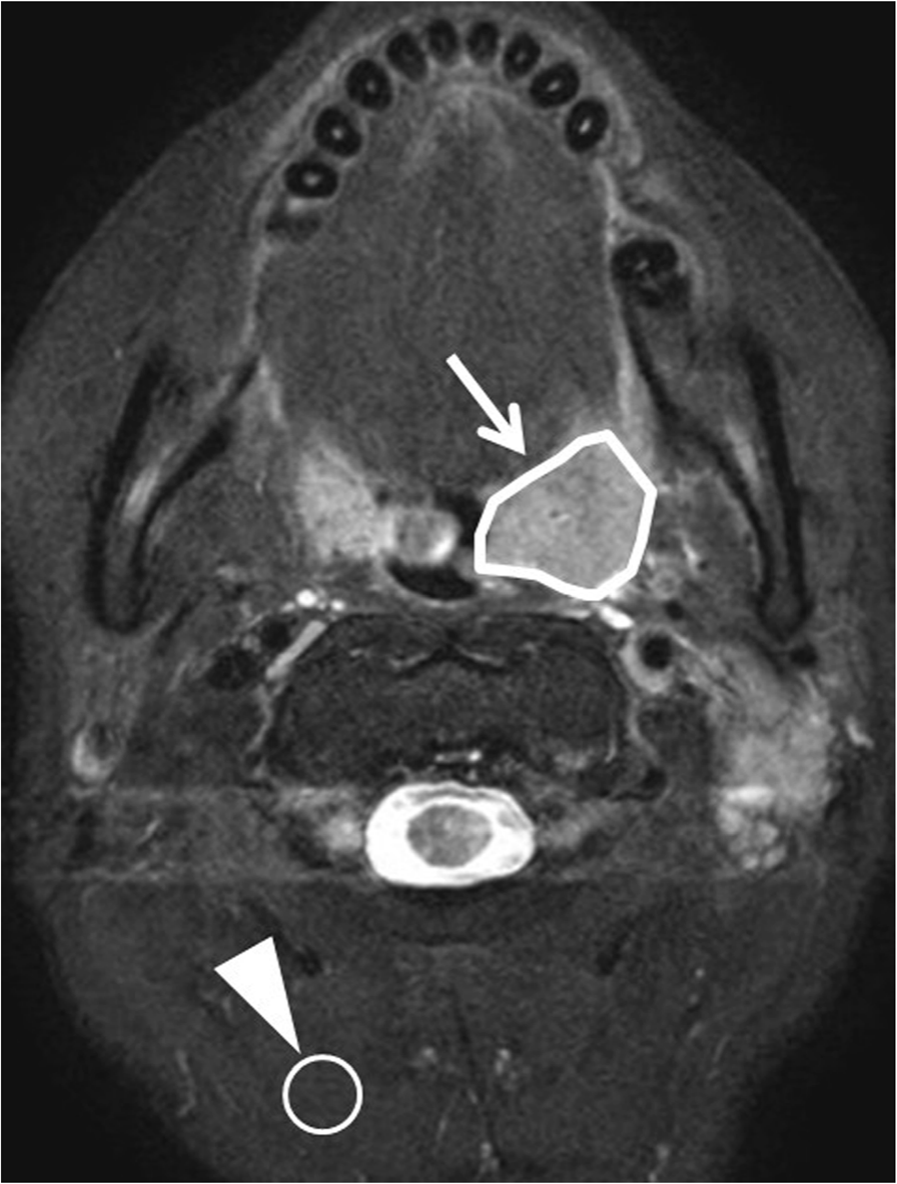
Tumor ROI delineation. The ROI was placed to delineate each primary site with a polygonal ROI on Fs-T2WI images. A round ROI (1 cm dia.) was also placed on the posterior neck muscle for the reference signal as background

The primary tumor was outlined by a board-certified neuroradiologist with 19 years of experience. It was performed on the axial Fs-T2WI with a polygonal region of interest (ROI). Any area which was suggested to be necrosis or a cystic lesion with very high signal intensity in Fs-T2WI was excluded from the ROI. If the tumor extended into two or more slices, the slice in which the largest area of tumor was depicted was selected. For the reference signal intensity estimation, a round ROI (1 cm dia.) was also placed on the posterior neck muscle for the reference signal as background, while avoiding the signal intensity of noise or artifact. Case example of ROI delineation was presented in Fig. 1. For ROI delineation on DWI, almost the same ROI was delineated on DWI b0 image with reference to the Fs-T2WI ROI so that the same region was delineated. Then this ROI was copied on a b1000 image.

#### Parameter calculation

Both histogram and texture analysis were performed by calculating the data within the ROI. The histogram features included the four commonly used parameters: the relative mean signal, the coefficient of variance (CV), kurtosis, and skewness. The texture analysis was performed using the gray-level co-occurrence matrix (GLCM), the most common and sensitive texture descriptor to calculate lesion heterogeneity in greater detail from the texture data [13]. The histogram parameter of relative mean signal value in each tumor was calculated as the mean value of the signal intensity in the tumor ROI, and by dividing the mean signal intensity of the outlined posterior neck muscle ROI. The histogram parameters of CV, kurtosis and skewness were calculated using the following equations [14]:

$$ CV=\sigma /\mu $$

$$ skewness=\left[1/n\ast \sum \left(\chi -{\mu}^3\right)\right]/{\sigma}^3 $$

$$ kurtosis=\left[1/n\ast \sum \left(\chi -{\mu}^4\right)\right]/{\sigma}^4-3 $$

where n is the number of pixels within the tumor ROI, *x* is the signal intensity value in each pixel, μ is the mean of *x*, and σ is the standard deviation of *x*. The CV describes the normalized measure of dispersion of signal intensity values. Histogram skewness describes the skew in the shape of the distribution curve of the signal intensity. The kurtosis describes the peak and/or flatness of the curve peak; a more acute peak has higher kurtosis, and a more broadened and/or flattened peak has lower kurtosis [14].

**Fig. 2:**
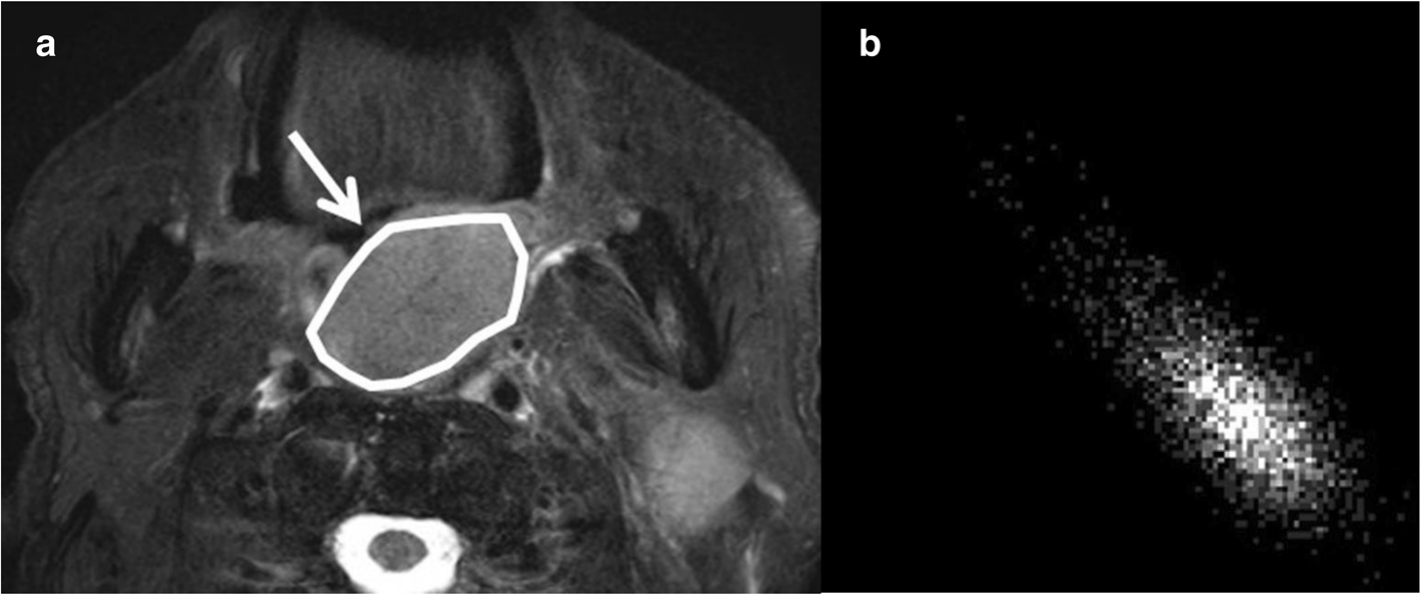
Example of GLCM data from tumor ROI. From the signal profile in the tumor ROI (**a**), the GLCM (**b**) was created, and then all GLCM parameter calculations were performed using all of the pixel data in the GLCM

The GLCM parameters were calculated using the following equations [15]:

$$ Contrast=\sum \limits_{i,j}{\left|p-j\right|}^2p\left(i,j\right) $$

$$ Correlation=\sum \limits_{i,j}\frac{\left(i-\mu i\right)\left(j-\mu j\right)p\left(i,j\right)}{\sigma_i{\sigma}_j} $$

$$ Energy=\sum \limits_{i,j}p{\left(i,j\right)}^2 $$

$$ Homogeneity=\sum \limits_{i,j}\frac{p\left(i,j\right)}{1+\left|i-j\right|} $$

where *p*(*i,j*) represents the (*i,j*) value of the GLCM. The GLCM features are spatially detailed information of signal intensity in the tumor ROI, compared to the histogram parameters (mean, CV, kurtosis and skewness). The GLCM is composed of the square plane with rows and columns from zero to the maximum value of the gray scale in tumor ROI. The GLCM element in row *i* and column *j* represents the number of times a given gray tone of value *i* is horizontally adjacent to gray tone *j* in the original quantized image. The GLCMs were calculated by using only directly adjacent pixels for simplicity. The details of GLCM feature were previously described [15], and example data are shown in Fig. 2. Finally, all parameters of relative mean signal, CV, kurtosis, skewness, contrast, correlation, energy and homogeneity were calculated in each tumor. In addition, for DWI analysis, the conventional apparent diffusion coefficient (ADC) was also calculated using 2 b-values (0 and 1000) signal data with following equation: (Signal intensity of b = 1000) / (Signal intensity of b = 0) = exp.(− 1000*ADC). The mean ADC value in the ROI was calculated in each SCC lesion. The calculation process was performed by using the self-developed program by MATLAB ver. 2012a (MathWorks, Natick, MA).

### Statistical analysis

For all obtained parameters, the correlation coefficients between all pairs of parameters were calculated using Pearson’s correlation coefficient. Correlation coefficients were classified as follows: r < 0.2, poor; *r* = 0.2–0.4, fair; *r* = 0.41–0.6, moderate; *r* = 0.61–0.8, good; *r* > 0.81, excellent.

We used the non-paired t-test to compare each parameter’s value between the total SCC patient group and the ML patient group, and between the two SCC histological grade subgroups (well/moderately differentiated SCC vs poorly differentiated SCC). In addition, a non-paired t-test was also performed to compare histogram and texture parameters between human papillomavirus (HPV) positive and negative oropharyngeal SCC cases. Before the non-paired t-test and the multiple comparison test, all obtained parameters were confirmed to be normally distributed by the Shapiro-Wilk test. *P*-values < 0.05 were considered significant. SPSS software (IBM, Armonk, NY) was used for all statistical analyses.

## Results

The detailed information and characteristics of the total series of 67 patients are summarized in Table 1. All parameters of the histogram and GLCM features were successfully calculated in all patients. Detail of all of the histogram and GLCM texture parameters among the three groups of well/moderately (*n* = 45) and poorly differentiated (*n* = 12) SCCs and MLs (*n* = 10) are summarized in Table 2. The correlation coefficients between each pair of obtained parameters are presented in Table 3. Notably, the contrast and the homogeneity of the parameters with GLCM features indicated a strong inverse correlation (*r* = − 0.93). All obtained parameters were confirmed to be normally distributed by the Shapiro-Wilk test.

**Table 1:** Patient characteristics (*n* = 67)

	Squamous cell carcinoma				Malignant lymphoma (*n* = 10)
	Well differentiated SCC (*n* = 24)	Moderately differentiated SCC (*n* = 21)	Poorly differentiated SCC (*n* = 12)	Total (*n* = 57)	
Age					
Range	49–81	48–80	48–80	48–81	37–83
Average	65.7	63.1	63.2	64.2	60.6
Gender					
Male	20	20	7	47	8
Female	4	1	5	10	2
Primary tumor site					
Oral cavity	9	8	6	23	1
Oropharynx	10	10	6	26	9
Hypopharynx	5	3	0	8	0
T-stage					
T1	0	0	0	0	–
T2	8	8	3	19	–
T3	9	6	5	20	–
T4	7	7	4	18	–
N-stage					
N0	7	8	4	19	–
N1	6	2	3	11	–
N2	11	10	5	26	–
N3	0	1	0	1	–
HPV status					
Positive	4	5	2	11	–
Negative	4	3	3	10	–
Unknown	16	13	7	36	–

**Table 2:** Detail of parameters among histological types in all patients

	Squamous cell carcinoma			Malignant lymphoma (*n* = 10)
	Well-/Moderately differentiated SCC (*n* = 45)	Poorly differentiated SCC (*n* = 12)	Total (*n* = 57)	
Histogram analysis				
Relative mean signal	3.85 ± 0.81	2.89 ± 0.63	3.65 ± 0.86	2.61 ± 0.49
Coefficient of variation (× 10^− 2^)	13.9 ± 3.2	11.3 ± 1.9	13.3 ± 3.1	11.2 ± 1.7
Kurtosis	0.52 ± 0.3	0.35 ± 0.34	0.48 ± 0.31	0.38 ± 0.18
Skewness	0.08 ± 0.41	0.05 ± 0.24	0.07 ± 0.37	−0.05 ± 0.17
GLCM Texture Feature				
Contrast	77.5 ± 13.9	56.2 ± 12.9	72.9 ± 16.2	49.3 ± 8.7
Correlation (×10^−2^)	7.63 ± 0.61	7.22 ± 0.44	7.55 ± 0.59	7.27 ± 0.41
Energy (× 10^−3^)	1.91 ± 0.55	2.04 ± 0.39	1.94 ± 0.52	1.72 ± 0.4
Homogeneity (× 10^−1^)	2.1 ± 0.18	2.56 ± 0.15	2.22 ± 0.25	2.53 ± 0.12

**Table 3:** The correlation coefficient of each pair among all parameters

	Relative mean signal	Coefficient of variation	Kurtosis	Skewness	Contrast	Correlation	Energy	Homo-geneity
Relative mean signal	–	0.15	0.28	0.08	0.66	0.27	0.46	−0.66
Coefficient of variation	–	–	0.37	0.17	0.25	0.7	−0.21	−0.26
Kurtosis	–	–	–	0.27	0.39	0.22	0.06	−0.39
Skewness	–	–	–	–	−0.14	0.2	−0.38	0.12
Contrast	–	–	–	–	–	0.26	0.32	−0.93
Correlation	–	–	–	–	–	–	−0.22	−0.19
Energy	–	–	–	–	–	–	–	−0.3
Homogeneity	–	–	–	–	–	–	–	–

In our comparison of the total SCC group and the ML patient group, the relative mean signal of the SCC patients (3.65 ± 0.86) was significantly higher than that of the ML patients (2.61 ± 0.49) (*p* < 0.01). The contrast in the SCC patients (72.9 ± 16.2) was also significantly higher than that of the ML patients (49.3 ± 8.7) (*p* < 0.01). The homogeneity in the SCC group (2.22 ± 0.25 × 10^− 1^) was significantly lower than that of the ML group (2.53 ± 0.12 × 10^− 1^) (*p* < 0.01). The CV tended to be lower in the ML group compared to the SCC group, but the difference was not significant (*p* = 0.061).

**Fig. 3:**
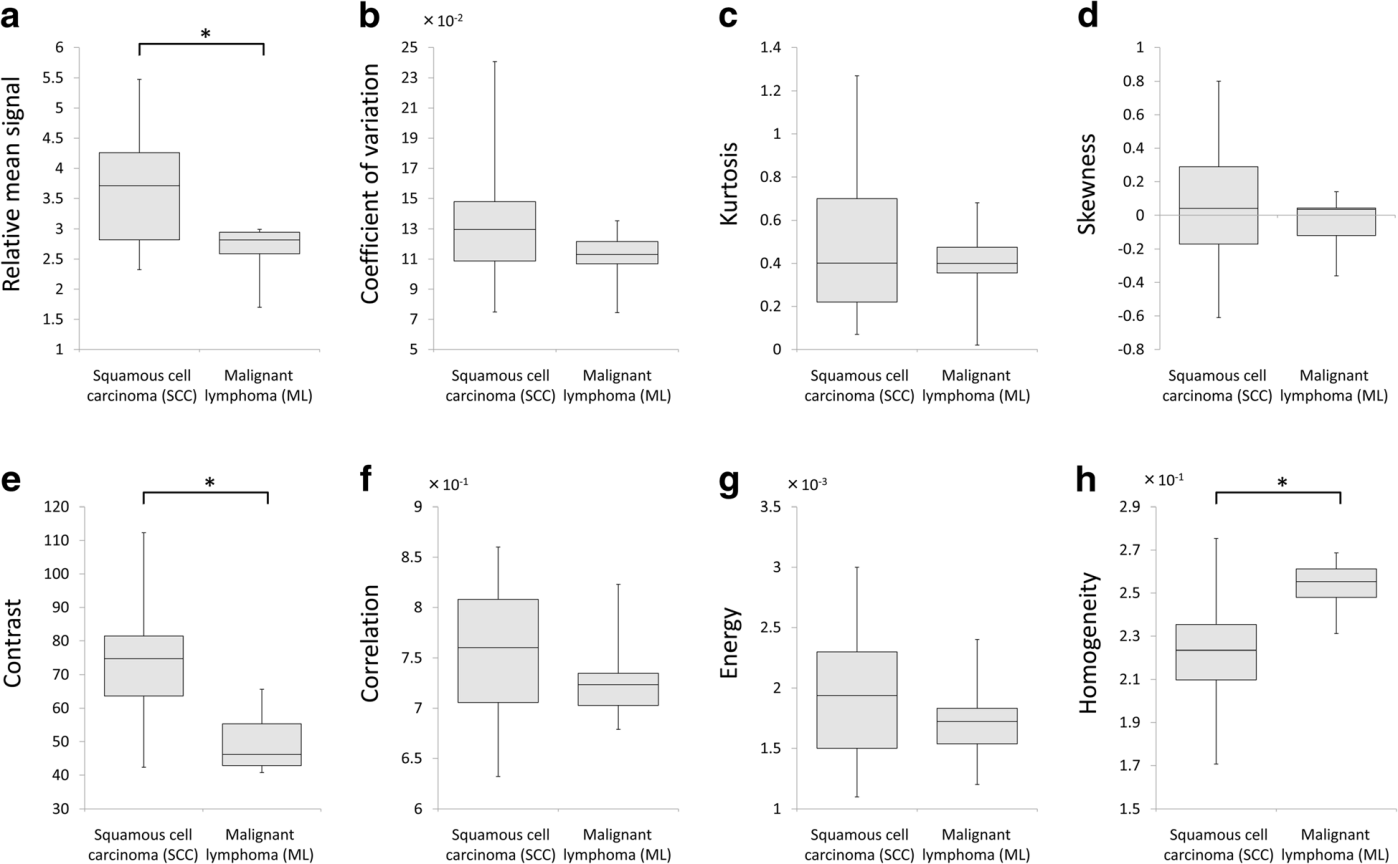
Histogram and GLCM texture parameters between the SCC and ML patients. Box-and-whisker plot for all histogram parameters (**a–d**) and GLCM texture parameters (**e–h**) in the total groups of SCC patients and ML patients were shown. Significant differences between the ML and SCC groups were observed in relative mean signal (a: *p < 0.01), contrast (e: *p < 0.01) and homogeneity (h: *p < 0.01). In addition, CV tended to be lower in the ML group (*p* = 0.061)

**Fig. 4:**
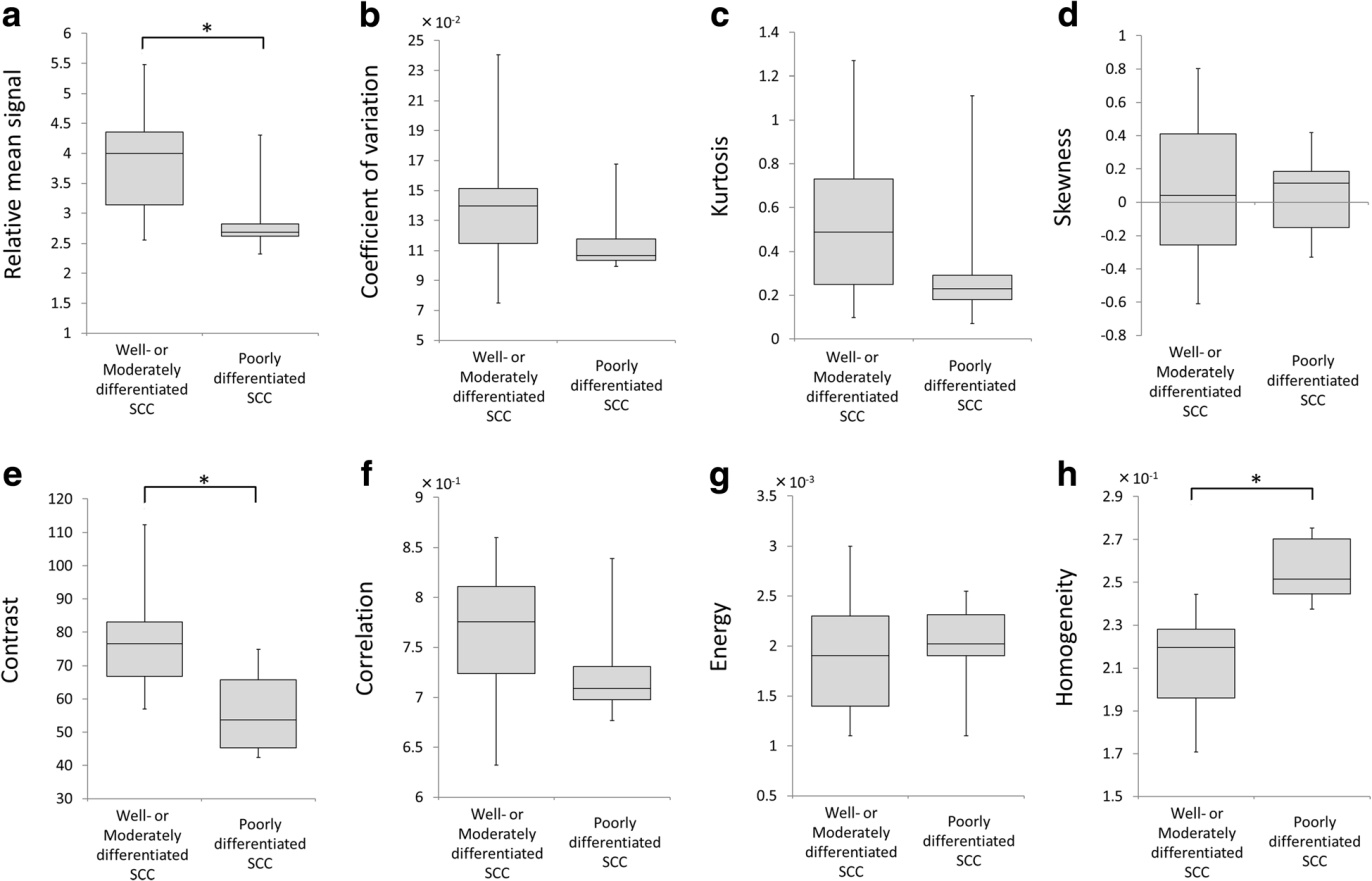
Histogram and GLCM texture parameters between the well/moderately and the poorly differentiated SCC patients. Box-and-whisker plots of all histogram parameters (**a–d**) and GLCM parameters (**e–h**) between the well/moderately and the poorly differentiated SCC patients were shown. Significant differences between the poorly differentiated SCC group versus the moderately and well differentiated SCC groups were observed in relative mean signal (a: *p < 0.01), contrast (e: *p < 0.01,) and homogeneity (h: *p < 0.001)

Between the well/moderately and the poorly differentiated SCC patients, the relative mean signal intensity was significantly lower in the poorly differentiated SCCs (2.89 ± 0.63) compared to the well/moderately SCCs (3.85 ± 0.81) (*p* < 0.01). The contrast values in the poorly differentiated SCCs (56.2 ± 12.9) were lower than those of the well/moderately SCCs (77.5 ± 13.9) (*p* < 0.01). The homogeneity in the poorly differentiated SCCs (2.56 ± 0.15 × 10^− 1^) was higher than the well/moderately SCCs (2.1 ± 0.18 × 10^− 1^) (*p* < 0.001). Each parameter’s box-and-whisker plot graph in the comparison between the SCC and ML patients is shown in Fig. 3 and the results of our comparison of the three histological grade groups of SCC patients are shown in Fig. 4.

In DWI analysis, the ADC values in the ML patient group (0.71 ± 0.1 × 10^− 3^ mm^2^/s) were significantly lower than those in the total SCC patient group (0.83 ± 0.12 × 10^− 3^ mm^2^/s) (*p* < 0.05). ADC values in poorly differentiated SCCs (0.77 ± 0.06 × 10^− 3^ mm^2^/s) tended to be lower than well/moderately differentiated SCCs (0.85 ± 0.12 × 10^− 3^ mm^2^/s) with nearly statistical significance (*p* = 0.07).

**Fig. 5:**
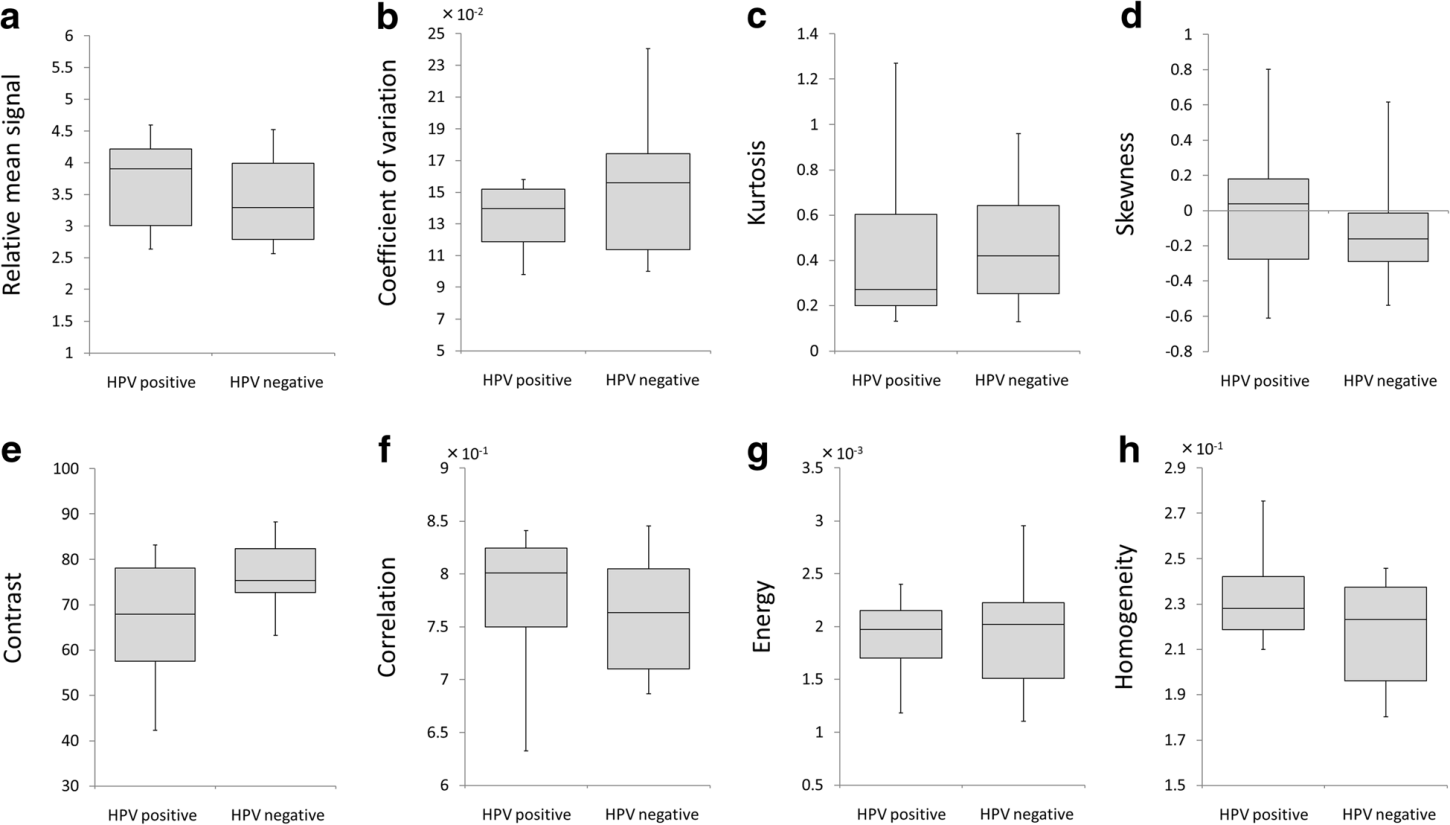
Histogram and GLCM texture parameters between HPV-positive and HPV-negative patients. Box-and-whisker plots of all histogram parameters (**a–d**) and GLCM parameters (**e–h**) between HPV-positive and HPV-negative patients were shown. The contrast tended to be lower in HPV-positive cases compared to -negative cases (*p* = 0.07). In addition, the homogeneity tended to be higher in HPV-positive cases than -negative cases (*p* = 0.09)

In the comparison of the histogram and texture parameters between HPV-positive and HPV-negative patients, the lower contrast (66.6 ± 14.5) and higher homogeneity (2.34 ± 0.23 × 10^− 1^) in HPV-positive cases compared to -negative cases (contrast: 76.8 ± 7.8, homogeneity: 2.17 ± 0.13) were revealed as nearly significant (*p* = 0.07, 0.09 respectively). Details of parameter values in the comparison of HPV status are summarized in Table 4 and Fig. 5.

**Table 4:** Detail of parameters in HPV positive and negative patients

	HPV Positive (*n* = 11)	HPV Negative (*n* = 10)
Histogram analysis		
Relative mean signal	3.65 ± 0.72	3.4 ± 0.72
Coefficient of variation (×10^−2^)	13.4 ± 2.2	15.2 ± 4.4
Kurtosis	0.42 ± 0.35	0.46 ± 0.28
Skewness	0.05 ± 0.43	−0.08 ± 0.4
GLCM Texture Feature		
Contrast	66.6 ± 14.5	76.8 ± 7.8
Correlation (×10^−2^)	7.79 ± 0.67	7.61 ± 0.56
Energy (×10^−3^)	1.89 ± 0.4	1.93 ± 0.55
Homogeneity (×10^−1^)	2.3 ± 0.22	2.17 ± 0.23

## Discussion

Using histogram and texture analysis by GLCM features, our study revealed significant differences in the relative mean signal, contrast and homogeneity in Fs-T2WI signal intensity between the SCC and ML patients, and also between the poorly differentiated SCC and well/moderately differentiated SCC patient groups. The relative mean signal result suggests that MLs tend to show lower Fs-T2WI signal intensity than SCCs, and similarly, poorly differentiated SCCs tend to show lower signal intensity than well/moderately differentiated SCCs. In addition, the contrast and homogeneity results revealed that the MLs showed lower contrast and more homogenous signal intensity in Fs-T2WI compared to the SCCs. Among our SCC cases, the poorly differentiated SCCs also showed lower contrast and more homogenous signal intensity values compared to the well/moderately differentiated SCCs. The MR texture analysis for head and neck lesions has been used in a very limited number of studies. Our present investigation provides the first report indicating the utility of the texture analysis for the differentiation of ML and the histological grades of SCC.

In our analysis of GLCM features, the contrast and homogeneity were revealed as significant parameters in the differentiation of MLs and SCCs, and also for the detection of poorly differentiated SCCs in a total SCC population. MLs and poorly differentiated SCCs have generally been observed to be homogeneous in the visual assessment of their imaging findings, and lymphomas in particular were described in several previous reports to have homogeneous signal intensity due to the intratumoral characteristics of high cellular density, a small amount of stromal tissue and less micro-necrosis [2, 16, 17]. Such findings can be quantitatively observed by GLCM features clearly. The CV also tended to be lower in our present group of MLs; this parameter also reflects the tumor homogeneity in Fs-T2WI. However, a significant difference was not observed. This result might mean that the detectability of homogeneous signal intensity was superior in the GLCM features compared to the histogram features. In contrast, imaging findings of a tumor homogeneous signal in Fs-T2WI can be also evaluated by experienced radiologists by visual assessment. However, the detection of this finding is sometimes difficult for radiologists who are not familiar with the interpretation of head and neck tumor imaging. Moreover, even for experienced radiologists, there are several cases in which the image interpretation differs between radiologists because of the differences in individual-based visual assessments. Compared to such individual imaging findings, the result of texture analysis can provide quantitative parameters that reflect the imaging findings in the tumor ROI. By using these quantitative parameters, objective assessments of the imaging findings can be achieved. We speculated that the in-plane signal complexity of tumor homogeneity can be successfully detected by the texture analysis by the GLCM features with a detailed calculation process compared to histogram parameters such as the CV. Although the two parameters of contrast and homogeneity showed significant differences, they were strongly inversely correlated with each other, with a correlation coefficient > 0.9. From this point of view, it appears that contrast and homogeneity might reflect almost the same characteristics, and could thus be used interchangeably.

We also observed that the relative mean signal intensity could be used for the differential diagnosis of ML and SCC, and for identifying poorly differentiated SCCs among a series of SCC patients. We speculated that this difference in the lower signal intensity by Fs-T2WI was also caused by the abovementioned issues (i.e., high cellular density, small amount of stromal tissue, and less micro-necrosis). Previous studies using DWI reported a lower ADC in ML and poorly differentiated SCC [18, 19]. The results of the present study also suggested the significantly lower ADC value in ML and the nearly significant lower ADC in poorly differentiated SCC, comparable with the abovementioned previous reports. These results may suggest the higher cellular density of ML or poorly differentiated SCC. The lower mean signal intensity might also reflect such intratumoral characteristics.

In the future, by combining the abovementioned parameters, the prediction of histological diagnosis can be achieved with a greater level of diagnostic accuracy as an additional supporting tool. The prediction of the histological information will be useful for the decision-making regarding the treatment strategy (e.g., surgical treatment, type of chemotherapy and radiotherapy). However, our present findings indicated that the parameters of relative mean signal, contrast and homogeneity were not largely different between poorly differentiated SCCs and MLs, because the signal profile in Fs-T2WI of these two types of cancer were similar to a degree. Further analyses should be performed using combinations of other MR-derived characteristics such as perfusion and diffusion parameters. Dynamic contrast-enhanced perfusion imaging can provide the perfusion-related parameters such as plasma volume fraction, extracellular extravascular volume fraction and vessel permeability; these may be useful as an additional tool. In addition, information gained from contrast enhancement enables more accurate tumor contouring by distinguishing the tumor tissue, necrosis, and cyst formation compared to the information obtained with T2WI only. The diffusion parameter of ADC which is considered to reflect the tumor microstructural environment is widely used for the evaluation of head and neck malignancy, especially in SCC [20], and the result of present study indicated its utility. Moreover, the recent techniques using a non-Gaussian-based diffusion model such as intravoxel incoherent motion or diffusion kurtosis imaging will provide greater detail regarding the tissue microstructure [21], and thus the parameters obtained by such diffusion models may also provide useful information. Adding these imaging parameters will contribute to the diagnostic accuracy with the combination of T2WI texture analysis data. The textural features of these diffusion and perfusion imaging modalities may be also useful. In addition, combinations of other modalities such as computed tomography or ^18^F-fluorodeoxyglucose positron-emission tomography with T2WI texture analysis data may also contribute to the diagnostic accuracy. The usefulness as an added value of these imaging techniques and modalities should be assessed in future studies.

This study has several limitations. First, the number of patients was small. With the analysis of a large number of patients, several tendencies can be observed in greater detail by dividing the patient population into groups by parameters such as the TNM stage. Such subgroup analyses may differentiate the tumor subtype more clearly with less overlap. In addition, a tendency of intratumoral less heterogeneity in HPV-positive cases was indicated by the textural parameters of contrast and homogeneity. Such a small number of patients may not be sufficient to detect significance in parameters. This tendency should be clarified by the analysis of a larger number of patients, because the HPV status is very important information that is related to tumor characteristics and the patient’s prognosis [22]. Second, we did not determine the reproducibility and inter-scanner reliability of parameters obtained by the histogram or GLCM feature analysis. The differences depending on the imaging parameters and image viewer were also not investigated. In particular, textural parameters can vary based on the type of MR equipment as well as imaging parameters, the post-process algorithm such as the imaging filter, and the imaging viewer. Parameter variations based on the abovementioned numerous influences should be further analyzed.

## Conclusion

Parameters of histogram and GLCM feature obtained by the texture analysis from Fs-T2WI may be useful for the determination of histological type and grade of head and neck malignancies. This information can be used as an additional supporting tool for the definitive diagnosis.

## Abbreviations

ADC: Apparent diffusion coefficient
CV: Coefficient of variance
DWI: Diffusion weighted imaging
Fs-T2WI: Fat-suppressed T2 weighted images
GLCM: Gray-level co-occurrence matrix
HPV: Human papillomavirus
ML: Malignant lymphoma
MRI: Magnetic resonance imaging
ROI: Region of interest
SCC: Squamous cell carcinoma
SPAIR: Spectral adiabatic inversion recovery
